# Pseudophakic bullous keratopathy

**DOI:** 10.22336/rjo.2017.17

**Published:** 2017

**Authors:** Stefan Pricopie, Sanziana Istrate, Liliana Voinea, Costin Leasu, Vanessa Paun, Ciuluvica Radu

**Affiliations:** * Ophthalmology Department, Bucharest Emergency University Hospital, Bucharest, Romania; ** Anatomy Department, “Carol Davila” University of Medicine and Pharmacy, Bucharest, Romania

**Keywords:** corneal disease treatment, corneal surgery, bullous keratopathy, cataract extraction

## Abstract

Pseudophakic bullous keratopathy is characterized by corneal stromal edema with epithelial and subepithelial bullae due to cell loss and endothelial decompensation through trauma during cataract surgery. Patients present decreased vision, tearing, and pain caused by ruptured epithelial bullae. Cataract affects approximately 20 million people worldwide, and this complication can occur in 1 to 2% of the cataract surgeries. This study reviewed the bullous keratopathy etiopathogenesis and the clinical and surgical treatment available for this corneal disease.

## Introduction

The cornea is a complex structure that is responsible for most of the refraction of the eye and, because of its highly exposed position, has a protective role, acting as a physical barrier to trauma and infection [**1**,**2**]. One of the most important property of the cornea is its transparency, which is a result of a number of factors: the absence of blood vessels, the regularity and smoothness of the covering epithelium, the regular arrangement of the extracellular and cellular components in the stroma, which is dependent on the state of hydration and metabolism of the stromal elements [**2**]. 

The cornea has one of the body’s highest density of nerve endings, with a subepithelial and a deeper stromal plexus, both supplied by the 1st division of the trigeminal nerve. This is the reason why disease processes like bullous keratopathy are associated with pain, photophobia, and reflex lacrimation [**1**].

**Corneal Pathophysiology**

The average central thickness of the normal adult human cornea is approximately 550µm for Caucasians and it remains constant between the second and sixth decades, but varies with the time of day and race [**3**]. 

The cornea consists of five layers from anterior to posterior: epithelium, Bowman’s layer, stroma, Descemet’s membrane, and endothelium. The composition of the stroma is not uniform; the anterior stroma contains a higher ratio of dermatan sulfate to keratan sulfate, making the posterior stroma more likely to swell with excess water in states of endothelial dysfunction [**4**].

Immunohistochemical studies showed deposits of a specific extracellular matrix component, such as fibrilin-1 which belongs to the family of extracellular matrix proteins associated with elastic microfibrils and tenascin-C, which is a glycoprotein that has great importance in healing and is found in the posterior collagen layer or in the subepithelial fibrotic areas of corneas with bullous keratopathy [**6**].

Growth factors and cytokines influence cell proliferation, inflammation, scarring, and fibrosis. Elevated levels of interleukin-2 (IL-2), interleukin-8 (IL-8), growth factor insulin (IGF-1), transforming growth factor (TGF- β) and bone marrow factor - 4 (BMP-4) were found in corneas with bullous keratopathy. The interactions between growth factors and extracellular matrix degrading matrix metalloproteins are important and can be a mechanism for the loss of corneal transparency [**6**].

Corneal deturgescence is maintained by endothelial cell sodium/ potassium-activated adenosine triphosphatase) and by tight junctions between the endothelial cells that limit the ingress of fluid. By removing fluid from the stroma and limiting its entry, endothelial cells maintain the ordered arrangement of collagen and preserve the cornea’s transparency. In states of deficient endothelial cell density, a lack of tight junctions between the endothelial cells allows the increased entry of fluid into the stroma. The endothelial cells that remain may have a higher concentration of Na+, K+-ATPase as a compensatory mechanism to increase fluid removal [**4**].

The normal endothelial cell density is greater than 3500 cells/ mm^2 in children and gradually declines with age to approximately 2000 cells/ mm^2 in older people, with an average of 2400 cells/ mm^2 for adults [**5**]. After this, the average cell loss is about 0.6 percent per year with the development of edema when the cell density drops below 700 cells/mm^2. 

**Etiopathogenesis**

The main cause of bullous keratopathy is the loss of endothelial cells due to surgical trauma, especially in cataract surgery at sixth decade patients, with or without lens implantation [**6**,**7**].

The localized increase of temperature associated with the phacoemulsification probe can lead to thermal damage to adjacent corneal tissue. Damage to the endothelium can be caused by high irrigation or aspiration rates that can result in turbulent flow with lens particles connected with it [**10**].

Also, the duration of phacoemulsification used during the surgery is very important because the ultrasound energy is associated with the production of free radicals, which are reactive species with one or more unpaired electrons in their outer orbits and can damage the corneal endothelium by oxidative stress [**10**].

Other etiologies include endothelial dystrophies such as Fuchs dystrophy, tumors of the anterior chamber such as myxoma, congenital abnormalities, like microcornea, acute and neovascular glaucoma, herpetic endotheliitis or surgeries that can lead to endothelial cell loss like trabeculectomy, intraocular lens scleral fixation, anterior chamber lens implants for aphakic correction and high ametropia, after argon laser, radial keratotomy [**6**].

Bullous keratopathy may occur in around 1 to 2% of the patients undergoing cataract surgery, which is about two to four million patients worldwide [**6**].

**Treatment options**

The **clinical treatment** for corneal edema should be based on topical hypertonic agents such as sodium chloride (5%), anti-inflammatory drugs, topical and/ or systemic antiglaucoma medications, because increased IOP can compromise endothelial cell function, corticosteroids, lubricants and sometimes, due to the pain experienced by the patients, therapeutic contact lenses to improve symptoms [**10**]. 

According to a study conducted in 2015, **systemic L-cysteine** facilitated corneal edema remission when administered in the postoperative period in patients after cataract surgery, thus advocating its concurrent use in patients developing bullous keratopathy.

An increased expression of several proinflammatory mediators at the protein level in the corneal epithelium was demonstrated in patients with pseudophakic corneal edema. These cytokines and MMP, which are a family of extracellular proteinases that degrade the extracellular matrix proteins, participate in the pathologic processes in the pseudophakic corneal edema and specifically contribute to the continuous degradation of Bowman’s layer and recurrent erosions of the corneal epithelium.

The MMPs have a pivotal role in a number of pathologic processes, including angiogenesis and wound healing, where matrix degradation takes place. MMP are activated by the “cysteine switch”. All modes of activation lead to a dissociation of Cys73 from the zinc atom with concomitant exposure of the active site.

Based on the presumption that high L-cysteine levels may act as regulatory substrate for MMPs, more studies should be conducted in order to establish the adjuvant role of systemic L-cysteine in pseudophakic bullous keratopathies [**7**].

The use of **conjunctival flaps** is effective but has been limited by its unacceptable cosmetic outcome [**6**].

**Corneal transplantation** is still the gold-standard treatment for bullous keratopathy patients, as it provides symptomatic relief and visual rehabilitation [**8**]. Some limitations such as visual acuity recovery occur because of the high astigmatism and, although the cornea is the most commonly transplanted tissue in the body and corneal grafts high success rate, there is also the risk of rejection [**6**,**9**].

**Penetrating keratoplasty** refers to a full thickness corneal transplant. In conventional posterior lamellar keratoplasty (LK) and the newer endothelial keratoplasty (EK) procedures, only the inner layers of the cornea are transplanted and there are multiple variants of this procedures that include deep lamellar EK, Descemet’s stripping (automated) EK (DSEK or DSAEK), Descemet’s membrane EK, and Descemet’s membrane automated EK [**11**]. 

The posterior lamellar keratoplasty technique requires surgical skill and hinders any necessary action in the anterior chamber, but it has the advantage of a lower risk of rejection and preservation of the receptor surface. It is a promising technique, but the endothelial cell loss is bigger than in penetrating keratoplasty [**6**].

In developing countries with a shortage of donor corneas and long waiting lists of patients awaiting corneal transplantation, patients need to be provided with relief of symptoms and, if possible, temporary improvement in vision [**12**].

**Corneal collagen cross linking (CXL)** with Riboflavin and ultraviolet A (UVA) radiations is a photochemical process that was introduced by Seiler and Spoerl at the University of Dresden for the treatment of corneal ectatic disorders such as keratoconus and post LASIK ectasias [**13**].

Corneal CXL is considered a new tool in the struggle for the temporary reduction in corneal edema in patients with bullous keratopathy. It has been found to improve corneal transparency, corneal thickness, and ocular pain after surgery [**12**].

The proposed mechanism of action is that riboflavin absorbs UVA light, which results in the production of free oxygen radicals. These highly reactive oxygen radicals then induce the cross-linking of corneal stromal collagen and strengthen the cornea [**13**].

Different studies showed that corneal CXL significantly improves corneal transparency, corneal thickness, and ocular pain one month postoperatively. This symptomatic relief probably resulted from CXL-induced stromal compaction and reduced bullae formation. However, it did not seem to have a long-lasting effect in decreasing pain and maintaining corneal transparency [**12**,**14**].

In 1999, Pires et al. successfully used **amniotic membrane (AM)** to control pain in patients with BK. They attributed their results to various protease inhibitors located in the stromal matrix of the AM, which are important for promoting epithelial healing and reducing stromal ulceration and inflammation [**16**].

AM facilitates re-epithelialization by providing a suitable substrate and a normal basement membrane, by promoting epithelial cell migration and adhesion. AM is also believed to produce several growth factors that support epithelial cells. When the amniotic membrane is applied to the cornea, keratocyte derived fibroblasts and myofibroblasts are known to migrate from the corneal stroma into the amniotic stroma. This contributes to the subepithelial fibrosis and also anchors the amnion epithelial sheet to the corneal surface [**15**].

Amniotic membrane transplant is effective in controlling pain in patients with pseudophakic bullous keratopathy and does not induce neovascularization, but is not the first treatment option because of the cost and needed time [**6**,**15**].

**Anterior stromal puncture (ASP)** is a simple and popular interventional option in the management of pseudophakic bullous keratopathy with low cost and rare complications [**6**,**15**,**17**].

Immunohistochemical studies have demonstrated an increased expression of extracellular matrix proteins important for the adhesion of basal epithelial cells such as fibronectin, laminin, and type IV collagen at stromal puncture sites. The secretion of these basement membrane components would increase the epithelial adhesion in the underlying stroma, which is associated with subepithelial fibrosis, thus creating a barrier to liquid penetration into the subepithelial space and decreased subepithelial bubble formation [**6**,**15**].

Hsu et al. were able to clinically correlate an improvement in pain symptoms with varying degrees of subepithelial fibrosis and epithelial attachment [**18**].

**Phototherapeutic keratectomy (PTK)** can improve pain by reducing corneal thickness and this would help the remaining endothelial cells maintain corneal hydration [**6**].

Several studies reported PTK to be elective in the management of patients with bullous keratopathy from a variety of etiologies; they reported that the bullae resolve and pain is abolished in a large proportion of patients treated with a superficial ablation [**19**,**20**].

The main sensory nerve plexus in the cornea, which is derived from the nasociliary branch of the ophthalmic division of the trigeminal nerve, is located in the stroma, in the immediately subepithelial region, with a lower density plexus deeper in the stroma [**19**]. The rationale for this treatment is the ablation of these nerve plexuses thereby reducing corneal sensation and, in addition, corneal scarring induces an increase of extracellular proteins such as laminin, fibronectin, type IV collagen and hemidesmosomes which promote a greater adhesion between the epithelium and stroma [**6**,**19**].

Deep PTK appears to be more successful in comparison with superficial PTK because of the increased scarring associated may also result in an increased stability of the epithelium and a deep ablation has a superior effect on decreasing pain by the ablation of the neural plexus in the cornea [**19**].

The same reasoning as in PTK is used also for **automated lamellar keratectomy** but, in this case, a traditional microkeratome is used for the removal of the corneal tissue. It is a fast procedure, which can be an important factor for some elderly patients who present difficulties in undergoing longer surgeries while remaining in dorsal decubitus [**6**].

## Conclusions

Although corneal transplantation is the treatment with the best results in improving pain and visual acuity, what has to be kept in mind is the research for alternative treatments for the patients with no visual potential or those who are waiting for an available cornea. For this group of patients, clinical therapy, use of therapeutic contact lens, systemic L-cysteine, anterior stromal puncture, conjunctival flaps, amniotic membrane transplantation, phototherapeutic keratectomy, and automated lamellar keratectomy can significantly improve the quality of life.
